# PP65 How Does The Spanish Health Technology Assessment Network Assess The Value Of Robotic Surgical Platforms? A Review Of Challenges Faced

**DOI:** 10.1017/S0266462324002368

**Published:** 2025-01-07

**Authors:** Beatriz Pellicer Vercher, María Álvarez Orozco, Carlos Mansilla Morales

## Abstract

**Introduction:**

The value assessment of robotic surgical platforms poses several challenges on existing health technology assessment (HTA) frameworks of robotic-assisted surgery (RAS). Following widely RAS adoption, this work aimed to assess current RAS evaluation from the Spanish HTA network perspective taking into consideration, in the absence of local guidance, the principles published by Erskine J et al. in 2023.

**Methods:**

A review of the HTA reports comparing RAS versus open surgery (OS) and laparoscopic surgery (LPS) conducted by the Spanish HTA network and published between 2017 and 2023 was performed and compared against the consensus statement on RAS evaluation set out around seven key themes (i.e., evidence inclusion and exclusion, patient- and clinician-reported outcomes [PRO, CRO, respectively]), the learning curve, allocation of costs, appropriate time horizons, economic analysis methods, and robotic ecosystem/wider benefits].

**Results:**

Four HTA reports, developed by AQuAS (Agència de Qualitat i Avaluació Sanitàries de Catalunya), assessing RAS versus OS and LPS were identified and reviewed in depth. Seven groups of indications and 57 outcomes were retrieved and classified according to the principles set out in the paper. Our findings show there is a strong lack of compliance with the consensus statement: real-world evidence studies are deprioritized or considered insufficient, and none of the reports provides either an economic evaluation or an assessment on the robotics ecosystem. Only PROs comply fully or partially, except for one report.

**Conclusions:**

Spanish HTA evaluation of robotic surgical platforms diverges significantly from the consensus. Discrepancies were observed across crucial domains, notably in evidence-selection criteria, economic evaluations, and holistic robotic ecosystem assessment, despite occasional compliance with PROs. Capturing specific, relevant outcomes to enable a comprehensive HTA beyond the context of therapy is key to informing decision-making and enhancing reliability of future evaluations.

